# Accumulation of intermediate denitrifying compounds inhibiting biological denitrification on cathode in Microbial Fuel Cell

**DOI:** 10.1186/s40201-015-0236-5

**Published:** 2015-11-24

**Authors:** Abdullah Al-Mamun, Mahad Said Baawain

**Affiliations:** Department of Civil & Architectural engineering, Sultan Qaboos University, Al-Khodh, P.C. 123, P.O. Box 33, Muscat, Sultanate of Oman; Centre for Environmental Studies and Research, Sultan Qaboos University, Al-khodh, P.C. 123, Muscat, P.O. Box 17, Muscat Sultanate of Oman

**Keywords:** Microbial fuel cell, Bio-cathode, Biological denitrification, Bioremediation, Process inhibition

## Abstract

**Background:**

Bio-cathode denitrifying microbial fuel cell (MFC) is a promising bio-electrochemical system (BES) where both the reactions of anodic oxidation and cathodic reduction are catalyzed by microorganisms. In this nitrogen removal process, a complete biological denitrification from nitrate (NO_3_^-^) to molecular nitrogen (N_2_) was achieved by four reduction steps, forming nitrite (NO_2_^−^), nitric oxide (NO) and nitrous oxide (N_2_O) as intermediate compounds. These enzymatic catalysis reductions are often slowed down on cathode electrode at the higher cathodic nitrate loading. This study investigated the cause for inhibition of the biological denitrification in a three-chambered MFC where the middle chamber acted as denitrifying bio-cathode and the two chambers at the side acted as bio-anode. Carbon fiber brushes were used as electrodes and nafion membranes were used as separator between the chambers.

**Results:**

The maximum power obtained was 14.63 W m^−3^ net cathodic compartment (NCC) (R_ext_ =11.5Ω) at an optimum nitrate loading of 0.15 kg NO_3_^−^-N m^−3^ NCC d^−1^. The accumulation of one of the intermediate denitrifying compound, e.g., NO_2_^−^ adversely affected biological denitrification rate on cathode. According to chemical kinetics, the accumulated NO_2_^−^ will form free nitrous acid (FNA, HNO_2_) in aqueous chemical system spontaneously. The study showed that approximately 45 % of the current production and 20 % of the total denitrification was decreased at a FNA concentration of 0.0014 ± 0.0001 mg HNO_2_ – N L^−1^ with an equivalent nitrite concentration of 6.2 ± 0.9 mg NO_2_^-^ - N L^−1^.

**Conclusions:**

The novel biological process indicates the potential of using denitrifying bio-cathode MFC for green energy production.

## Background

**Fig. 1:**
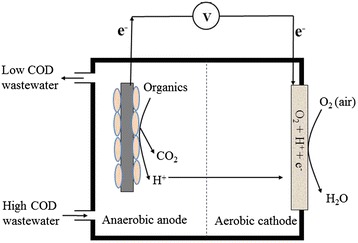
Principle of MFC technology

Microbial fuel cell (MFC) could be a sustainable hybrid technology for removal of bio-degradable organics and generation of electricity from various types of pure organic substrates and wastewater [1, 2]. In an MFC, the biochemical reactions are catalyzed by electrogenic bacteria on the anaerobic anode surface and the electrons and protons are produced from the degradation of organics. Concurrently, another set of bio-electrochemical reactions are taken place in the aerobic cathode surface, whereby the anode produced electrons and protons are consumed for reduction [3–6]. Fig. 1 shows the electron harvesting procedure in an MFC. A typical MFC comprises of an anaerobic anode and an aerobic cathode separated by a proton exchange membrane (PEM) or by an electrolytic solution (wastewater) by which the protons diffuse from the anode toward the cathode. The flow of electrons and the potential difference between the respiratory enzymes of anodic microbes and the oxygen reduction reaction on cathode generates current and voltage, respectively [7, 8].

In MFC, the most commonly used electron acceptor for cathodic reaction is oxygen due to its abundant availability, higher redox potential and eco-friendly character [9]. Rather than oxygen, some other chemical electron acceptors such as Fe (III), Mn (ΙV), and ferricyanide or bio-reducing substance such as NO_3_^−^, SO_4_^2−^ have been used by different researchers [10–14].

The added benefits of treating wastewater in MFC compared to that of the conventional activated sludge process are – (a) no requirement of aeration for COD removal, which reduces the power consumption; (b) direct conversion of substrate into electricity without any intermediate steps; (c) low generation rate of sludge; (c) generated electricity can be used to operate other units of the treatment plant [10, 15, 16]. Despite the advantages, the MFC technology still faces serious limitations in terms of large-scale application due to the use of costly catalyst (Pt) coating on cathode electrode and the lack of cost-effective proton exchange membrane or the knowledge of optimum operating conditions [17]. Due to these limitations, a number of issues have to be resolved through research and development before having this technology as a cost-effective alternative for green energy production. Among them, the major concerns are the selection of cheaper electrode materials [18] and the optimization of the reaction processes in order to maximize electrical power output and reduce installation costs. An MFC operating with a cost-effective bio-cathode would hence be of major interest for practical application of this technology for wastewater treatment. This research investigates the potential application of denitrifying bio-catalyst on cathode electrodes as an eco-friendly alternative of the costly Pt catalyst in MFC.

**Fig. 2:**
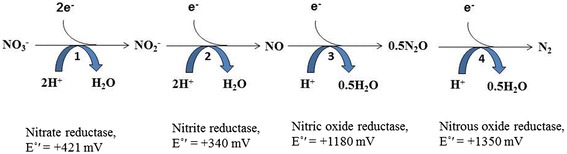
Biochemistry of biological denitrification with redox potential (E°′)

Denitrifying bio-cathode MFC is a complete bio-electrochemical system (BES) where both the anodic oxidation and the cathodic reduction reactions are catalyzed by microorganisms [19]. The complete biological denitrification from nitrate (NO_3_^−^) to molecular nitrogen gas (N_2_) is a step down reduction reaction forming nitrite (NO_2_^−^), nitric oxide (NO) and nitrous oxide (N_2_O) as intermediate compounds. The biochemistry details of the biological denitrification process are shown in Fig. 2 with the redox potential values and the name of the enzymes at each step [20]. Several studies on biological denitrification with environmental media, such as soil and sediments have demonstrated that the N_2_O reductase is inhibited at high oxygen and NO_2_^−^ concentrations or at low pH [21–23]. The inhibition of the N_2_O reductase will lead to the accumulation of intermediate denitrifying compounds and decrease the total rate of biological denitrification.

There are several mechanisms involved to inhibit the biological denitrification process. That is why, the dominating mechanism of inhibition to biological denitrification is still ambiguous. One study on the biological nitrogen removal from wastewater have reported that the accumulation of nitrite (10 mg NO_2_^−^- N L^−1^) is a possible cause of N_2_O release from denitrifying sludge [24]. Some other studies have speculated that the enzymatic competition between the terminal electron acceptors is a cause of inhibition to biological denitrification [25]. Kinetic studies on N_2_O reductase using pure bacterial cultures have demonstrated that the activity of nitrous oxide reductase enzyme is not only dependent on accumulated NO_2_^−^ concentration, but also on the pH of the media [26]. According to chemical kinetics, the accumulated NO_2_^−^ will spontaneously form free nitrous acid (FNA) in the aqueous chemical system depending on pH of the aqueous media [27]. Incorporating the concept of FNA generation inside the NO_2_^−^ containing aqueous solution, some recent studies on biological denitrification have claimed that FNA inhibits the growth and energy generation into a variety of phylogenetic organisms, including denitrifiers, denitrifying poly-phosphate accumulating organisms [28, 29], ammonia and nitrite oxidizing bacteria [30]. However, all of those studies investigated the inhibitory effects of NO_2_^−^ as well as FNA only on heterotrophic denitrifying microorganisms. None of the previous study has investigated the inhibitory effects of NO_2_^−^ and FNA on autotrophic denitrifying microbes. The main goal of this study is to have an improved understanding of any possible inhibition of the accumulated intermediate denitrifying compounds that takes place on the autotrophic denitrifiers, which act as biocatalysts on the cathode surface of MFC.

The study was carried out to (i) *understand the behavior of autotrophic denitrifying microbes under various cathodic nitrate loading for electricity production in MFC*, (ii) optimize cathodic nitrate loading rate using synthetic acetate solution as electron donor for denitrification, (iii) determine the attainable electricity and power production based on this cathodic denitrification, (iv) determine the rate of nitrogen removal in cathode chamber, and (v) *determine the optimal conditions for the bio-cathode denitrification without inhibition of the intermediately formed denitrifying compounds.*

## Materials and methods

### Design of MFC

**Fig. 3:**
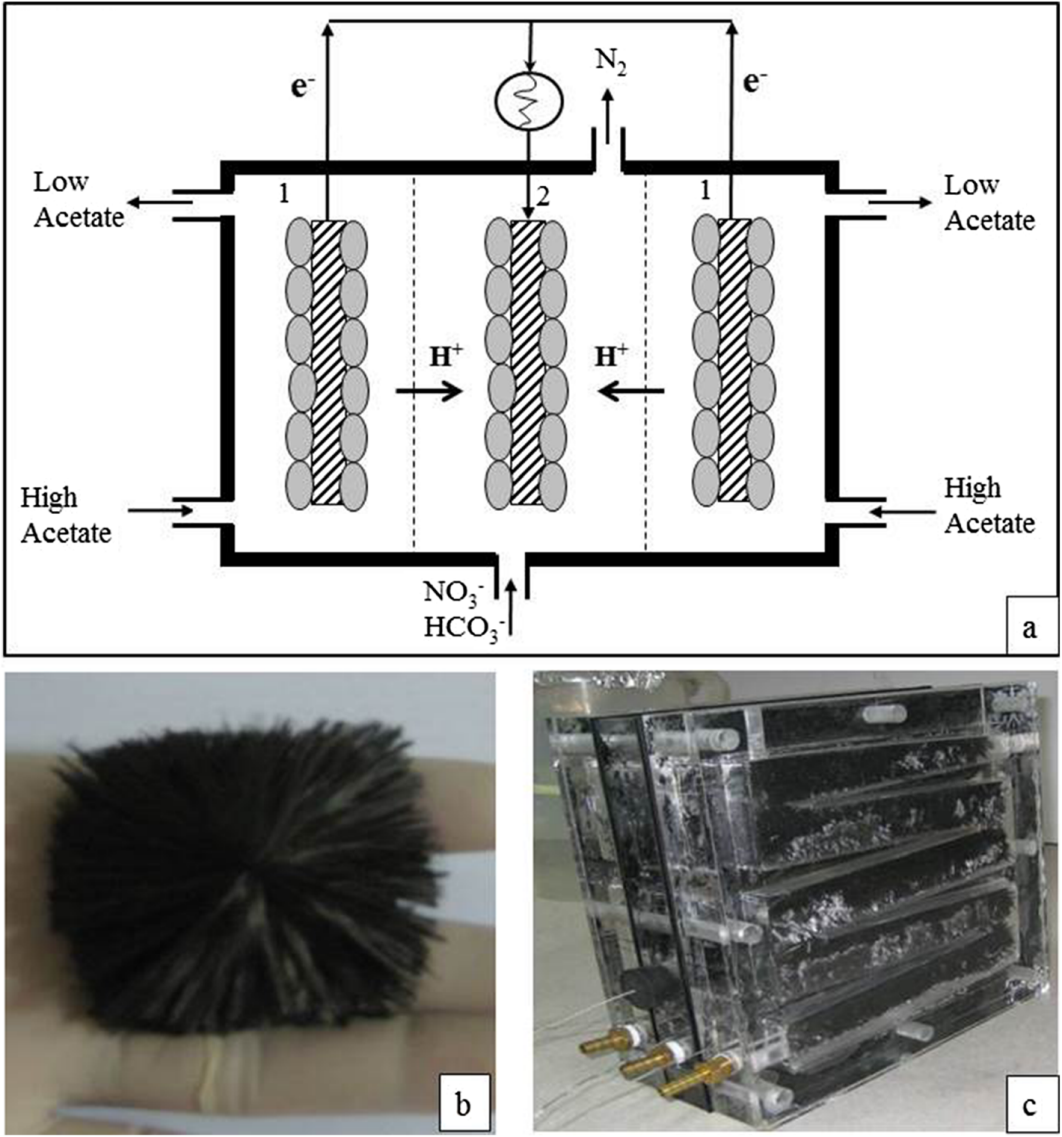
**a** Bio-cathode denitrifying MFC system: (1) anode chamber, (2) cathode chamber; **b** The cross-sectional view of the carbon brush electrode; and **c** The whole assembly of the MFC

The autotrophic denitrifying bio-cathode MFC system used in this study was illustrated in Fig.3a showing the flow directions of anodic and cathodic liquid streams, and bio-chemical species. The MFC contained three up-flow baffle-channeled chambers. In the assembled MFC, the middle chamber acted as denitrifying bio-cathode and two chambers at the sides acted as bio-anode. The bacteria grown on the anode and cathode electrode surfaces as attached biofilm catalyzed the anodic and cathodic reactions. Acetate containing synthetic wastewater continuously pumped through the anodic chambers, where bacteria utilized the organic substrate in its metabolic pathways to produce CO_2_, protons and electrons. The electrons produced at the metabolic process were collected at the anode electrode and diverted towards the cathode electrode, where autotrophic denitrifying bacteria retrieved them to reduce NO_2_^-^ to N_2_ gas. A synthetic wastewater containing nitrate was continuously pumped into the cathode chamber by a peristaltic pump (master flex, USA). Due to a proton motive force, the produced protons in the anode chamber migrated to the cathode surface by means of diffusion within the electrolytes and throughout the cation exchange membrane.

The actual internal design of the baffled channel in each chamber was shown in Fig. 3c. Each channel had a 3-cm square uniform cross-sectional area along the entire length. The midline length of each channel was 0.9 m. The channel was made by thorough cutting of a 3-cm thick acrylic plate with CNC machine. The horizontal portions of the channel were inclined at 4.1° upward direction to facilitate the up-ward movement of produced gas (biogas in anode chamber and N_2_ gas in cathode chamber) inside the channel. The internal volume of channel in each chamber was 0.75 × 10^−3^ cubic meter.

Both the anode and the cathode chambers were filled with a long and square cross-sectional fiber brush electrode. The brush electrode was made of carbon fibers (PANEX35 CONTINUOUS TOW, 50 K, ZOLTEK) with the following properties: tensile strength of 3.8 × 10^6^ KPa, Tensile Modulus of 241 × 10^6^ KPa, and Density of 1.81 g cm^−3^. The carbon fiber bunches were cut to a designated length and wound using an industrial brush manufacturing system into a twisted core consisting of two stainless steel wires (wire diameter of 0.5 mm). Then the circular cross sectional brush was trimmed to a 3 cm square cross-sectional brush (Fig. 3b) using a hair dressing electric razor so that the brush was completely filled in the 3 cm square cross-sectional up-flow channel. Prior to use, the carbon fibers were treated with ammonia gas as described by Logan et al. [31, 32]. The mass of the carbon fibers used in each channel was 26 ± 1.5 g.

According to the mass of fibers used in each channel of the MFC and an average fiber diameter of 7.2 μm, the estimated brush surface area was 7.98 ± 0.46 m^2^ or 10,650 ± 600 m^2^ m^−3^ of the reactor volume at an attained brush packing density of 7 % in the reactor. The volume of the total cathodic chamber (TCC) is 0.75 L and the liquid volume after packing of brush in the channel, the net cathodic chamber (NCC), was 0.69 L. The total volume of the two anodic chambers (TAC, total anode chambers) was 1.5 L and the volume of the net anodic chamber (NAC) was 1.38 L. Both the anode and cathode chamber was separated by a proton exchange membrane (nafion-112, 2 mill thickness, Gashub Technology). The pretreatment of the PEM was done by sequentially boiling it at 80 °C in H_2_O_2_ (3 %), deionized water, 0.5 M H_2_SO_4_ and then deionized water, each for 1 h and then stored in deionized water prior to being used [33].

### Bacterial inoculation

Both the anodic and the cathodic liquid streams were circulated (1.25 × 10^−3^ L min^−1^ or 0.075 L h^−1^) in an upflow mode for inoculation. In the anode chambers, sieved sewage (355-μm diameter sieve, effluents from primary settling tank, Sultan Qaboos University wastewater treatment plant, Oman) was circulated continuously as a substrate and inoculums during the start-up period by a peristaltic pump (Master flex, USA). The characteristics of domestic wastewater averaged 312 ± 32 mg L^−1^ of total COD, 92 ± 13 mg L^−1^ of soluble COD, 217 ± 64 mg L^−1^ of SS, 195 ± 56 mg L^−1^ of VSS, 405 ± 5 mg L^−1^ of TDS, 38 ± 4 mg L^−1^ of TN and 39 ± 34 mg L^−1^ of PO_4_^3−^. The pH was maintained at 7.2 ± 0.1 (these data shows averages and standard deviations based on a series of 4 samples).

At inoculation stage, the cathodic liquid stream was recirculated in up-flow mode by an external recirculation vessel. The cathodic liquid streams were consisted of a modified buffer medium. The detail composition of the buffer medium [34] and the trace minerals and vitamins solution [35–37] were collected from previous studies on bacterial culturing procedure. Different types of aerobic and anaerobic sludge (activated sludge, digester sludge) and sediments (sediments from primary and secondary settling basins) were mixed in order to obtain cathodic inoculums (2.5 mL of dewatered sludge in 1 L of cathodic liquid stream) with sufficient microbial diversity. A concentrated KNO_3_ solution was added into the recirculation vessel twice a day to achieve a desired volumetric loading rate of 0.1 kg NO_3_^−^- N m^−3^ net cathodic compartment (NCC) d^−1^ (33.33 mg NO_3_^−^-N L^−1^ of buffer solution pumped at a flow rate of 1.25 × 10^−3^ L min^−1^ or 0.075 L h^−1^).

### Operational conditions

After inoculating the anode electrode with domestic wastewater, the anodic streams were switched to a synthetic acetate solution containing the same modified buffer medium (the buffer medium used in cathode chamber) with sodium acetate. During this operational period, the sole electron donor for the anodic microbes was acetate and the sole electron acceptor for the cathodic microbes was nitrate. The concentration of nitrate and acetate for different cathodic nitrate loading are mentioned in Table 1. Each specific nitrate loading was fed approximately 30 days, keeping the constant feeding of acetate solution at the anodic chamber at an stoichiometric ratio of 2.5 (Acetate carbon/Nitrate nitrogen).

**Table 1:** Operating conditions when synthetic nitrate and acetate solution were used

^a^Cathodic nitrate loading (kg NO_3_^−^- N m^−3^ NCC d^−1^)	^a^Acetate concentration in anodic liquid (mg NaAc L^−1^)	Anodic and cathodic liquid flow rate (L min^−1^)	Operating resistances (Ω)	pH of the cathodic liquid stream
0.05	96	2 × 10^−3^	10.5	7.0 ± 0.10
0.1	192	2 × 10^−3^	10.5	7.0 ± 0.10
0.125	240	2 × 10^−3^	10.5	6.7 ± 0.1
0.15	286	2 × 10^−3^	10.5	6.3 ± 0.2
0.2	381	2 × 10^−3^	10.5	6.0 ± 0.1
0.25	450	2 × 10^−3^	10.5	5.8 ± 0.1

^a^Acetate loading to anode chamber was 2.5 times higher than that needed as stoichiometric requirement for nitrate reduction in cathode chamber (stoichiometric ratio of Acetate carbon/ Nitrate nitrogen = 2.5)

### Calculations

Cell potential (E_*cell*_, V) was measured with a multimeter connected to a computer by a data acquisition system (PC1604, TTi, RS) at every 30 min interval. Power (*P*, W) was calculated as: *P* = *I · E*_*cell*_, where current (*I*, A) was determined according to the ohm’s law: *I = E*_*cell*_*/ R*_*ext*_*,* and *R*_*ext*_ (Ω) is the fixed external resistance. Volumetric power (*P*_*ν*_, W m^−3^ NCC) was determined by *P*_*ν*_ = *E*^*2*^_*cell*_ / (*V · R*_*ext*_), where *V* (m^3^) is the net volume of cathodic compartment.

The open circuit voltage (OCV) of an MFC was the maximum cell potential generated by the system under infinite resistance (no current). Polarization and power densities were obtained by varying the external circuit resistance from infinity to 1 Ω using a resistor box (RS-201 precision resistance substitute, IET LABS, INC). The cell potential values were recorded only after the pseudo-steady-state conditions had been established. The establishment of this pseudo-steady-state might took several minutes or more, depending on the cathodic nitrate concentration and the external resistance. By changing the external resistance, we obtained a new cell potential, and hence a new current density and power density.

According to Anthonisen et al. [27], the spontaneous generation of FNA was calculated by the concentration of accumulated nitrate (NO_2_^−^) formed as intermediate compounds in the biological denitrification process, pH, and temperature as follows:

1 $$ \mathrm{F}\mathrm{N}\mathrm{A}\ \mathrm{a}\mathrm{s}\ {\mathrm{HNO}}_2\left({\mathrm{mg}\ \mathrm{L}}^{-1}\right)=\frac{46}{14}X\frac{N{O}_2^{-}-N\ \left( mg\ {L}^{-1}\right)}{K_a\ X\ {10}^{-pH}} $$

In which, K_a_ is the ionization constant of the nitrous acid equilibrium equation and it is also varied with temperature [27]. The value of K_a_ is related to temperature (°C) by

2 $$ {K}_a = {e}^{- \frac{2300}{\left(273 + {}^{\circ}\ C\right)}} $$

### Analytical methods

The concentration of NO_3_^−^, NO_2_^−^, PO_4_^3−^, NH_4_^+^ and SO_4_^2−^ in the anodic and cathodic liquid stream were determined by an Ion Chromatograph (DIONEX-500 fitted with GP50 Gradient pump and CD20 conductivity detector) with IonPac CS12A cation and IonPac AS9-HC anion column. In those measurements, samples were first filtered through a 0.2-μm pore sized membrane before analysis. Acetate was analyzed using a gas chromatograph (Shimadzu, AOC-20i) equipped with a FIT detector and a 25 m × 0.32 mm × 0.5 μm HP-FFAP column. Samples were also filtered through a 0.2 μm pore sized membrane and acidified using formic acid before analysis. Produced N_2_ gas analyses were performed using a gas chromatograph (GC-17A, Shimadzu) with charlston 80/100 porapak column using Helium gas as carrier. Total nitrogen was measured using a Shimadzu TNM-1 unit coupled with a TOC-V analyzer. In both case, samples were pre-filtered through a 0.2 μm pore sized membrane.

## Results and discussion

### Effect of different cathodic nitrate loading on current and power generation of MFC

**Fig. 4:**
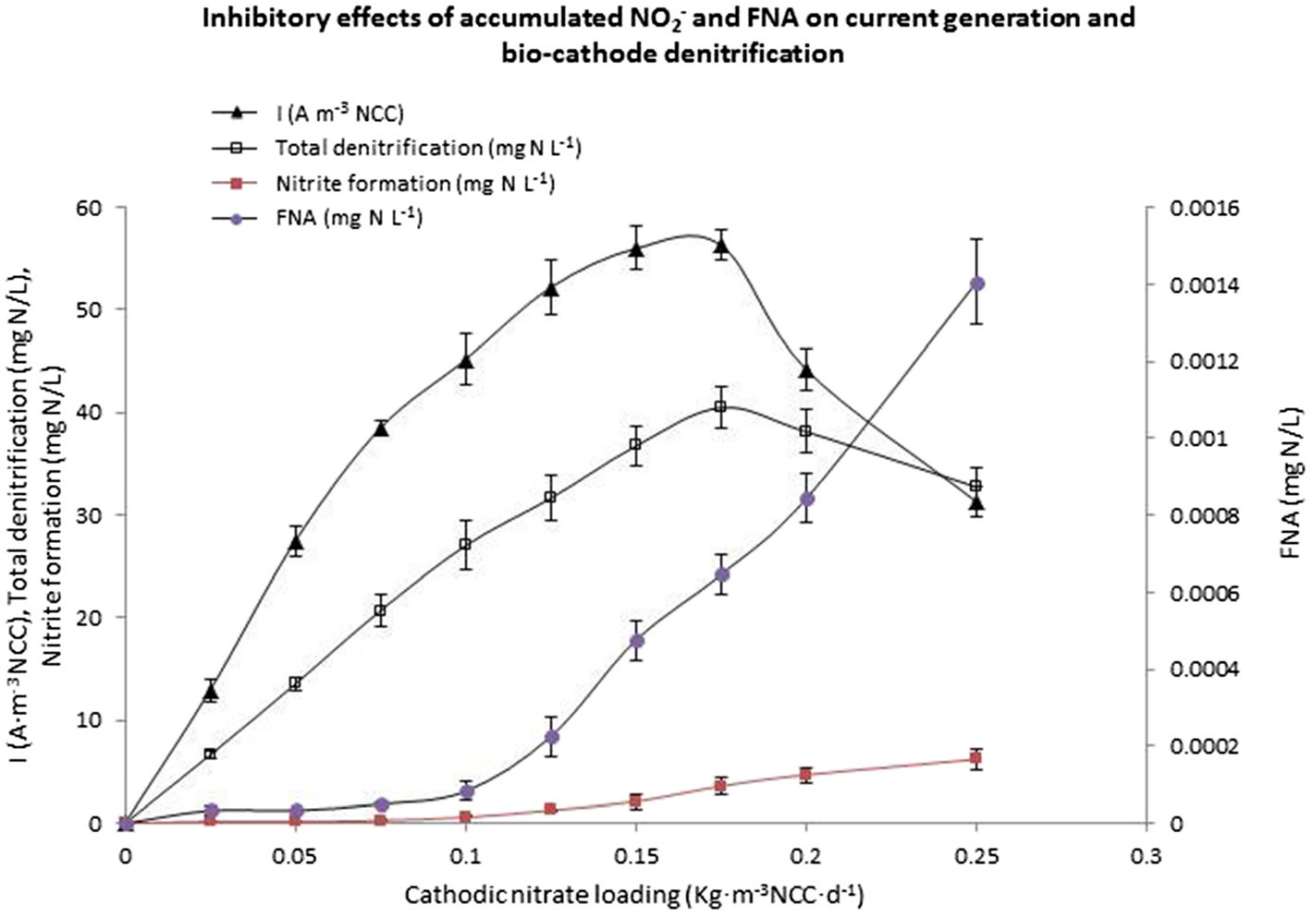
The inhibitory effect of intermediate denitrifying products, nitrite as well as FNA on the current generation and bio-cathode denitrification using 10.5 Ω fixed external resistance (Results showing as averages and standard deviations of 3 samples)

Two similar set of MFCs were operated simultaneously for better understanding of the results. During the operational period, both the MFCs were operated with the synthetic acetate solution as the anodic influent and the synthetic nitrate solution as the cathodic influent. The MFCs were operated at different cathodic nitrate loadings using 10.5 Ω as external resistance. Each combination of substrate loading (i.e., nitrate in cathode chamber and acetate in anode chamber) was operated for 30 days. During each combination of substrate loading, the current production and the denitrification rate was increased gradually with the increase of microbial population inside the MFC reactor and subsequently, reached to a saturated value. The saturation conditions were achieved by approximately 18 to 20-day continuous feeding of substrate at every specific substrate loading. Here the recorded current production and denitrification rates were the saturated values for each specific substrate loading. Fig. 4 showed the saturated current generation profiles with the gradual increase of specific cathodic nitrate (substrate for the cathodic denitrifying bacteria) loading from 0.05-0.25 kg NO_3_^−^- N m^−3^ NCC d^−1^. The maximum saturated current production obtained in this bio-cathode MFC system at an external resistance of 10.5 Ω was 53.8 ± 1.6 A m^−3^ NCC with 0.15 kg NO_3_^−^- N m^−3^ NCC d^−1^ of cathodic nitrate loading. The current production rate was slightly higher than that found by Virdis et al., 2008, 2009 [19, 38].

The current generation profiles showed that the rate of current production was decreased instead of increasing at a cathodic nitrate loading of more than 0.15 kg NO_3_^−^- N m^−3^ NCC d^−1^. A detailed study on why current production was decreased at higher cathodic nitrate loading was investigated by monitoring the accumulation of possible intermediate denitrifying compounds. Fig. 4 showed the inhibitory effect of the intermediate denitrifying product - nitrite (NO_2_^−^), which was formed during the denitrification and current generation process of the denitrifying bio-cathode MFC. The formation of nitrite is associated with the formation of free nitrous acid (FNA, the protonated form of nitrite ion, HNO_2_) in the aqueous chemical system spontaneously. A recent study revealed that the formation of FNA was enhanced at the higher acidic conditions of the aqueous system [39]. The similar findings were also observed in this study. Table 1 showed that the pH values of the cathodic liquid stream were neutral (pH = 7.0 ± 0.10) at lower nitrate concentrations and it was dropped gradually for the nitrate concentration of more than 0.1 kg NO_3_^−^- N m^−3^ NCC d^−1^. These data confirmed that the acidity of the cathodic liquid stream was increased with the increase of the cathodic nitrate-loading rate for more than 0.1 kg NO_3_^−^- N m^−3^ NCC d^−1^. Fig. 4 showed that the formation of FNA increased exponentially at nitrate concentration of more than 0.1 kg NO_3_^−^- N m^−3^ NCC d^−1^. Therefore, the total understanding of these two data confirmed that the increased acidity level of the cathodic liquid stream at higher cathodic nitrate loading rate was enhanced the exponential formation of FNA. The formation of nitrite (NO_2_^−^, denitrifying product) at the lower cathodic nitrate-loading rate did not show any inhibition to the microbial process, but the formation of FNA at higher cathodic nitrate loading inhibited the microbial process on cathode surface. These findings demonstrated that the observed degree of inhibition correlated much more strongly with the FNA, rather than nitrite concentration, indicating FNA as the true inhibitor on the activity of denitrifying microbes. Similar inhibitory effects of FNA were found to impart a wide range of phylogenetic types of bacteria, including both ammonia and nitrite oxidizing bacteria [27, 30, 40], denitrifiers and also denitrifying enhanced phosphorus removal organisms [28, 41]. The results showed that both the current generation and the denitrification activity were decreased at a cathodic nitrate loading rate of more than 0.175 kg NO_3_^−^-N m^−3^ NCC d^−1^. Approximately 45 % of the current production and 20 % of the denitrification activity in this bio-cathode MFC was decreased at a FNA concentration of 0.0014 ± 0.0001 mg HNO_2_-N L^−1^ (equivalent to the nitrite concentration of 6.2 ± 0.9 mg NO_2_^−^-N L^−1^ at a pH of 7 ± 0.1). The results showed that the autotrophic denitrifying bacteria is more tolerant than that of the heterotrophic denitrifying bacteria as the FNA concentration of 0.0014 ± 0.0001 mg HNO_2_-N L^−1^ caused only 20 % decrease of the autotrophic denitrification in the present study, whereas the FNA concentration of 0.0007-0.001 mg HNO_2_-N L^−1^ caused 50 % decrease of the heterotrophic dentrification [41].

### Polarization and Power generation

Table 2 shows that the maximum power production increased gradually with a gradual increase of initial cathodic nitrate loading. But the initial cathodic nitrate loading of more than 0.15 kg NO_3_^−^-N m^−3^ NCC d^−1^ could not produce higher power. This is due to the inhibitory effect by the intermediately formed denitrifying compounds (nitrite and FNA) to the denitrifying organisms on bio-cathode.

**Table 2:** Maximum OCV, current and power obtained at different cathodic nitrate loadings

Cathodic nitrate loading (kg NO_3_^−^-N m^−3^ NCC d^−1^)	Maximum power (W m^−3^ NCC)	Maximum OCV (V)	External R at max^m^power (Ω)
0.050 (20.83 mg NO_3_-N L^−1^)	6.92	0.492	18.5
0.100 (20.83 mg NO_3_-N L^−1^)	9.56	0.523	18.5
0.125 (26.05 mg NO_3_-N L^−1^)	13.52	0.599	11.5
0.150 (31.25 mg NO_3_-N L^−1^)	14.63	0.624	11.5
0.200 (41.67 mg NO_3_-N L^−1^)	11.79	0.603	14.5

The maximum volumetric power obtained was 14.63 W · m^−3^ NCC (*R*_*ext*_ = 11.5 Ω) at a cathodic nitrate loading of 0.15 kg NO_3_^−^-N m^−3^ NCC d^−1^, indicating the potential of using denitrifying bio-cathode MFC for energy production. The obtained maximum power was approximately 83 % higher than that obtained by Clauwaert et al. [34]. The higher maximum power obtained was due to the special internal design of the MFC reactor that achieves a plug flow condition. The cathodic nitrate loading beyond 0.15 kg NO_3_^−^-N m^−3^ NCC d^−1^ could not produce more power indicated the limitations of acetate as electron donor for biological denitrification.

## Conclusions

In this study, a bio-cathode MFC was investigated to achieve complete biological denitrification on cathode surfaces by the help of electrons supplied from the organics oxidation during synthetic wastewater treatment at anode surfaces. The prime goal of this study was to explore the feasibilities of a continuous up-flow baffled channel MFC using carbon fiber brush electrode for treating synthetic wastewater in anode and biological nitrogen removal (denitrification) in cathode with attainable electricity and power production. The results of the experiments performed over 6 months of operation with acetate demonstrated the feasibilities of combined carbon and nitrogen removal process with electrical energy recovery. The optimum cathodic nitrate loading corresponds to the electrons supplied from the COD removal from synthetic acetate solution was 0.15 kg NO_3_^−^-N m^−3^ NCC d^−1^ indicating that the bio-cathode denitrification is relatively dependent on the electron supplying capacity of the anodic substrate. Hence, the anodic substrate has a limit to act as electron donor for this type of autotrophic denitrification. At the same time, the MFC reactor design and the selection of materials should be optimized to achieve sufficient microbial density at the vicinity of the MFC electrode to harvest maximum electrons. The highest volumetric power obtained was 14.63 W m^−3^ NCC with concurrent denitrification of 148.3 ± 1.4 g N m^−3^ NCC d^−1^. This power generation rate was significantly higher than that found by the similar studies using granular graphite electrode, indicating the good internal design of this MFC reactor.

The initial nitrate loading to the cathodic bio-film has an effect on the denitrification and current generation process. The inhibitory effect of the intermediate denitrifying product- nitrite (NO_2_^−^) as well as FNA was observed. The current production and the denitrification activity of this bio-cathode MFC system was reduced approximately 45 % and 20 % respectively, at a FNA concentration of 0.0014 ± 0.0001 mg HNO_2_-N L^−1^ (equivalent to the nitrite concentration of 6.2 ± 0.9 mg NO_2_-N L^−1^ at a pH of 7 ± 0.1). However, this study shows the practical relevance of a novel denitrification process using MFC even though it has not been fully optimized at this stage. Future investigation might be required to understand the reductase enzyme activity for the step down biological denitrification and the possible electron competitions for the reductase enzyme under respective redox potential.

## Abbreviations

MFC: Microbial fuel cell
BES: Bio-electrochemical system
NCC: Net cathodic compartment
FNA: Free nitrous acid
TCC: Total cathodic compartment
TAC: Total anodic compartment
NAC: Net anodic compartment
OCV: Open circuit voltage

## References

[1] Kim BH, Kim HJ, Hyun MS, Park DH (1999). Direct electrode reaction of Fe (III))-reducingbacterium Shewanella putrefaciens. J Microbiol Biotechnol.

[2] Rabaey K, Clauwaert P, Aelterman P, Verstraete W (2005). Tubular microbial fuel cell for efficient electricity generation. Environ Sci Technol.

[3] Bond DR, Holmes DE, Tender LM, Lovley DR (2002). Electrode-reducing microorganisms that harvest energy from marine sediments. Science.

[4] Liu H, Logan BE (2004). Electricity generation using an air-cathode single chamber microbial fuel cell in the presence and absence of a proton exchange membrane. Environ Sci Technol.

[5] Lefebvre O, Shen Y, Tan Z, Uzabiaga A, Chang IS, Ng HY (2011). Full-loop operation and cathode acidification of a microbial fuel cell operated on domestic wastewater. Bioresour Technol.

[6] Lefebvre O, Nguyen TT, Al-Mamun A, Chang IS, Ng HY (2010). T-RFLP reveals high β-Proteobacteria diversity in microbial fuel cells enriched with domestic wastewater. J Appl Microbiol.

[7] Logan BE, Hamelers B, Rozendal R, Schröder U, Keller J, Freguia S (2006). Microbialfuel cells: methodology and technology. Environ Sci Technol.

[8] Lefebvre O, Al-Mamun A, Ooi WK, Tang Z, Chua DH, Ng HY (2008). An insight into cathode options for microbial fuel cells. Water Sci Technol.

[9] Tran HT, Ryu JH, Jia YH, Oh SJ, Choi JY, Park DH (2010). Continuous bioelectric-ity production and sustainable wastewater treatment in a microbial fuel cell constructed with non-catalyzed granular graphite electrodes and permeable membrane. Water Sci Technol.

[10] Rabaey K, Lissens G, Siciliano SD, Verstraete W (2003). A microbial fuel cell capable of converting glucose to electricity at high rate and efficiency. Biotechnol Lett.

[11] Rabaey K, Ossieur W, Verhaege M, Verstraete W (2005). Continuous microbial fuel cells convert carbohydrates to electricity. Water Sci Technol.

[12] Rhoads A, Beyenal H, Lewandowski Z (2005). Microbial fuel cell using anaerobic respiration as an anodic reaction and biomineralized manganese as a cathodic reactant. Environ Sci Technol.

[13] Shantaram A, Beyenal H, Raajan R, Veluchamy A, Lewandowski Z (2005). Wireless sensors powered by microbial fuel cells. Environ Sci Technol.

[14] Lefebvre O, Al-Mamun A, Ng HY (2008). A microbial fuel cell equipped with a biocathode for organic removal and denitrification. Water Sci and Tech.

[15] Jang JK, Pham TH, Chang IS, Kang KH, Moon H, Cho KS (2004). Construction andoperation of a novel mediator- and membrane-less microbial fuel cell. Process Biochem.

[16] Clauwaert P, Aelterman P, Pham TH, Schamphelaire LD, Carballa M, Rabaey K (2008). Minimizing losses in bio-electrochemical systems: the road to applica-tions. Appl Microbiol Biotechnol.

[17] Li Z, Yao L, Kong L (2008). Electricity generation using a baffled microbial fuel cell convenient for stacking. Bioresour Technol.

[18] Lefebvre O, Ooi WK, Tang Z, Al-Mamun A, Chua D, Ng HY (2009). Optimization of a Pt-free cathode suitable for practical applications of microbial fuel cells:. Bioresour Technol.

[19] Virdis B, Rabaey K, Yuan Z, Rozendal RA, Keller J (2009). Electron fluxes in a microbial fuel cell performing carbon and nitrogen removal. Environ Sci Technol.

[20] B.H. Kim and G.M. Gadd, Bacterial Physiology and Metabolism, Cambridge University Press, 2007, pp. 302–303.

[21] Fujita K, Dooley DM (2007). Insights into the mechanism of N_2_O reduction by reductively activated N_2_O reductase from kinetics and spectroscopic studies of pH effects. Inorg Chem.

[22] Schulthess RV, Kuhni M, Gujer W (1995). Release of nitric and nitrous oxides from denitrifying activated sludge. Water Res.

[23] Shiskowski DM, Mavinic DS (2006). The influence of nitrite and pH (nitrous acid) on aerobic-phase, autotrophic N_2_O generation in a wastewater treatment bioreactor. J Environ Eng Sci.

[24] Itokawa H, Hanaki K, Matsuo T (2001). Nitrous oxide production in high-loading biological nitrogen removal process under low COD/N ratio condition. Water Res.

[25] Dendooven L, Erson JM (1994). Dynamics of reduction enzymes involved in the denitrification process in pasture soil. Soil Biol Biochem.

[26] Gorelsky SI, Ghosh S, Solomon E (2006). Mechanism of N_2_O reduction by the μ(4)-S tetranuclear Cu-z cluster of nitrous oxide reductase. J Am Chem Soc.

[27] Anthonisen AC, Loehr RC, Prakasam TBS, Shinath EG (1976). Inhibition of nitrification by ammonia and nitrous acid. J Water Pollut Control Fed.

[28] Zhou Y, Pijuan M, Yuan ZG (2007). Free nitrous acid inhibition on anoxic phosphorus uptake and denitrification by poly-phosphate accumulating organisms. Biotechnol Bioeng.

[29] Zhou Y, Ganda L, Lim M, Zhiguo Y, Taffan K, Ng WJ (2010). Free nitrous acid (FNA) inhibition on denitrifying poly-phosphate accumulating organisms (DPAOs). Appl Microbiol Biotechnol.

[30] Vadivelu VM, Yuan Z, Fux C, Keller J (2006). The inhibitory effects of free nitrous acid on the energy generation and growth processes of an enriched nitrobacter culture. Environ Sci Technol.

[31] Logan B, Cheng S, Valerie W, Garett E (2007). Graphite fiber brush anodes for increased power production in air cathode microbial fuel cells:. Environ Sci Technol.

[32] Cheng S, Logan BE (2007). Ammonia treatment of carbon cloth anodes to enhance power generation of microbial fuel cells:. Electrochemical Communications.

[33] Ghasemi M, Wan Daud WR, Ismail M, Rahimnejad M, Ismail AF, Leong JX (2013). Effect of pre-treatment and biofouling of proton exchange membrane on microbial fuel cell performance. Int J Hydrog Energy.

[34] Clauwaert P, Rabaey K, Aelterman P, De Schamphelaire L, Ham TH, Boeckx P (2007). Biological denitrification in microbial fuel cells. Environ Sci Technol.

[35] Balch WE, Fox GE, Magrum LJ, Woese CR, Wolfe RS (1979). Methanogens: reevaluation of a unique biological group. Microbiol Rev.

[36] Lovley DR, Greening RC, Ferry JG (1984). Rapidly Growing Rumen Methanogenic Organism That Synthesizes Coenzyme-M And Has A High-Affinity For Formate. Appl Environ Microbiol.

[37] Lovley DR, Phillips EJP (1988). Novel mode of microbial energy metabolism: organic carbon oxidation coupled to dissimilatory reduction of iron or manganese. Appl Environ Microbiol.

[38] Virdis B, Rabaey K, Yuan Z, Keller J (2008). Microbial fuel cells for simultaneous carbon and nitrogen removal. Water Res.

[39] Jiang G, Gutierrez O, Yuan Z (2011). The strong biocidal effect of free nitrous acid on anaerobic sewer biofilms. Water Res.

[40] Vadivelu VM, Yuan Z, Fux C, Keller J. Stoichiometric and kinetic characterization of nitrobacter in mixed culture by decoupling the growth and energy generation processes: Biotechnol. Bioeng. 2006 10.1002/bit.20956 16673416

[41] Zhou Y, Pijuan M, Zeng RJ, Yuan Z (2008). Free nitrous acid inhibition on nitrous oxide reduction by a denitrifying enhanced biological phosphorus removal sludge. Environ Sci Technol.

